# Letters of Welcome

**DOI:** 10.1186/1617-9625-1-4-229

**Published:** 2003-12-15

**Authors:** Gary Doer, Glen Murray, Dave Chomiak

**Affiliations:** 1 Legislative Building, Winnipeg, Manitoba, Canada, R3C OV8

## A message from the Premier

It is with great pleasure that I extend a warm welcome on behalf of the citizens and government of the Province of Manitoba to all those participating in the International Society for the Prevention of Tobacco Induced Diseases Scientific Annual Meeting.

Tobacco related diseases are maladies that touch many people. Too many friends and loved ones have been lost to smoking and tobacco consumption. It is more important than ever, in this time of dramatically rising health care costs, that we work together to prevent sickness and diseases however we can. This is particularly true of our young people. Manitoba has, in fact, implemented laws prohibiting smoking in order to provide minors a truly smoke-free environment and a better example for how to stay healthy.

We were very pleased to learn that our capital city of Winnipeg had been chosen to host this important meeting of scientific minds from around the world. I know all the delegates will learn first-hand why we have been chosen by an increasing number of organizations to host their conferences and meetings. We boast many world-class amenities, a host of entertaining and enjoyable things to do and see, as well as deliciously diverse cuisine that is the envy of jurisdictions many times our size. Still, I think you will find that the thing that we are known best for is having the friendliest people on earth.

I would like to take this opportunity to welcome you and to wish you well in your meetings and deliberations.

Gary Doer

## A message from Mayor Glen Murray

It is with real admiration and respect that I extend greetings to each and every one of you as you partake in the International Society for the Prevention of Tobacco Induced Diseases' Scientific Annual Meeting.

The caliber of knowledge amassed at this Meeting is incredible and I know you will have a successful and informative session while in our City.

For those of you visiting from outside the Winnipeg area, I hope you will have a chance to explore some of our many attractions. Winnipeg offers wonderful river walks and a host of activities and restaurants offering cuisine from every corner of the globe.

Again, on behalf of my colleagues on City Council and all citizens of Winnipeg, welcome and best wishes.

GLEN MURRAY,

MAYOR

## A message from Dave Chomiak

To Conference Delegates:

It is my pleasure to welcome the International Society for the Prevention of Tobacco Induced Diseases (PTID) to Winnipeg, Manitoba for its Scientific Annual Meeting in September 2003.

Manitoba Health is firmly committed to tobacco control. In January 2002, our government announced a comprehensive provincial strategy for tobacco control that focuses on youth, and adheres to the four strategic goals of the national strategy: prevention, protection, cessation and de-normalization. Additionally, as part of the provincial strategy, we have amended legislation to prohibit retailers from displaying tobacco and tobacco related products where they may be visible to children. These provisions came into effect on January 1, 2004. It is my understanding that Manitoba and Saskatchewan are the only two jurisdictions in the world to restrict the display of tobacco products in this manner.

Manitoba Health looks forward to the outcomes and findings of your Annual Meeting as we continue to plan tobacco control initiatives here in Manitoba.

I wish you every success with your Annual Meeting, and I hope that you have a pleasant and productive stay in Winnipeg.

Sincerely,

Dave Chomiak

cc Dr. David A. Scott

Ms Rachelle Normand

## 

Please see additional files 1 and 2.

## Competing interests

The authors declare that they have no competing interests.

## Supplementary Material

Additional file 1 Click here for file

Additional file 2 Click here for file

