# Supplementary material for: Letters of Welcome
Source: Tob Induc Dis. 2003 Dec 15;1(4):229–31. doi: 10.1186/1617-9625-1-4-229 (PMC2672095; doi:10.1186/1617-9625-1-4-229)
Supplement: Additional file 1 [file 1617-9625-1-4-229-S1.pdf]

*The International Society for the*
*Prevention of Tobacco Induced Diseases*

**International Scientific Conference**

*Tobacco Induced Diseases: Challenges for the 21st Century*

Winnipeg, Canada
September 29 to October 1st, 2003

# LIST OF PRE-REGISTERED DELEGATES AND SCIENTIFIC CONTRIBUTORS

| | NAME | COUNTRY | ABSTRACT # |
|---|---|---|---|
| 1 | Deb Adleman | Canada | 38 |
| 2 | Ayad Alsagheer | Canada | |
| 3 | Roddy Andoh | Ghana | |
| 4 | Catalena Birek | Canada | 5 |
| 5 | Jennifer Borden | Canada | 22, 39 |
| 6 | Reuben Botchway | Ghana | |
| 7 | Julia Broderick | Canada | |
| 8 | Douglas Brothwell | Canada | 38 |
| 9 | George Brown-Otoo | Ghana | 42, 43 |
| 10 | David S Celermajer | Australia | 26 |
| 11 | Shu W Chan | USA | 26 |
| 12 | Ping Chook | Hong Kong | 26 |
| 13 | Perry Chowdhury | USA | 24,40 |
| 14 | John E. Connett | USA | 12 |
| 15 | Tom Czyczko | Canada | 46 |
| 16 | Michael E. DeBakey | USA | 17 |
| 17 | Nykola Dubenski | Canada | 21 |
| 18 | Nicole Dunn | Canada | 19, 36 |
| 19 | Fortune Eboyem | | |
| 20 | Jiang Zheng Feng | China | 26 |
| 21 | Norm Fleming | Canada | |
| 22 | Valentive Galitovsky | USA | 40 |
| 23 | Shirley Gelskey | Canada | 37, 38, 439 |
| 24 | Murray Gibson | Canada | |
| 25 | Jean-Michel Halimi | France | |
| 26 | Wojciech Hanke | Poland | 34 |
| 27 | Anthony J Hedley | Hong Kong | 2 |
| 28 | Eberhard Heissen | Germany | |
| 29 | Akiko Higashiyama | Japan | 20 |
| 30 | Xin Huang | China | 26 |
| 31 | Kristina Hunter | Canada | 21 |
| 32 | Allan Hynes | Canada | 35 |
| 33 | Teruo Inoue | Japan | |
| 34 | Toru Kato | Japan | 16 |

| | | | |
|---|---|---|---|
| 35 | Leah Kells | Canada | 37 |
| 36 | Alexander R. Kerr | USA | |
| 37 | Julia Klein | Canada | 4 |
| 38 | Denis Kinane | USA | 14 |
| 39 | Asuka Komatsu | Japan | 18 |
| 40 | Gideon Koren | Canada | 31 |
| 41 | Tricia Kutnikoff | Canada | 21 |
| 42 | Anna Kwiatek | Poland | |
| 43 | Ernie Lam | Canada | 6 |
| 44 | Tai H Lam | Hong Kong | 2 |
| 45 | Philip Lazarus | USA | 7 |
| 46 | Morley Lertzman | Canada | 21 |
| 47 | Hok C Leong | Macau | 26 |
| 48 | Paula G. Lindgren | USA | 12 |
| 49 | Kan-Zhi Liu | Canada | 3, 35 |
| 50 | Dan Longo | USA | 29 |
| 51 | Andrew Loughead | Canada | 46 |
| 52 | Angela Man | Canada | 35 |
| 53 | Fumiko Matsuda | Japan | 18 |
| 54 | Paul W. McDonald | Canada | 1 |
| 55 | Sarah McGhee | Hong Kong | 2, 13 |
| 56 | Hideshi Miura | Japan | 20 |
| 57 | Kazuaki Miyagishima | Japan | 18 |
| 58 | Sherry Mooney | Canada | 19, 36 |
| 59 | Suzanne Moore | UK | 15 |
| 60 | David J. Murphy | USA | 10 |
| 61 | Robert Murray | Canada | 12, 21 |
| 62 | Anke Nelson | Germany | |
| 63 | Toshitaka Nakahara | Japan | 18 |
| 64 | Megumi Noami | Japan | 18 |
| 65 | John W. Norris III | USA | 10 |
| 66 | Malcolm Ogborn | Canada | |
| 67 | Caroline Pampolina | Canada | |
| 68 | P.B. Patel | | |
| 69 | Jean-Pierre Picard | Canada | 37, 39 |
| 70 | Kinga Polanska | Poland | 34 |
| 71 | Peter YK Poon | Hong Kong | 26 |
| 72 | Vijay Pruthi | Canada | |
| 73 | Mui Qiao | Hong Kong | 26 |
| 74 | Lars Ramström | Sweden | 11 |
| 75 | Dennis Ranalli | USA | 9 |
| 76 | Babak Rashidkhani | Iran | 23 |
| 77 | Kazunari Satomura | Japan | 18 |
| 78 | Kevin Saunders | Canada | 19, 36 |
| 79 | David Singer | Canada | 35, 37, 39 |
| 80 | David Scott | Canada | 22, 35, 37, 39 |
| 81 | Elliott Scott | Canada | 33 |
| 82 | Rana Sidhu | Canada | |
| 83 | Steve Sussman | USA | 28 |

| | | | |
|---|---|---|---|
| 84 | Gay Sutherland | UK | 27 |
| 85 | Yuko Takahashi | Japan | 20 |
| 86 | Mark Taylor | Canada | 21 |
| 87 | G. Neil Thomas | Hong Kong | 26 |
| 88 | Kodethoor Udupa | USA | 24 |
| 89 | Pawar Vilas | India | 45 |
| 90 | Guo G Wang | China | 26 |
| 91 | Jiang Wang | USA | 17 |
| 92 | Xing Li Wang | USA | 17 |
| 93 | Saman Warnakulasuriya | UK | 8 |
| 94 | Erica White | Canada | 19, 36 |
| 95 | Kam Sang Woo | Hong Kong | 26 |
| 96 | Nancy Yu | Canada | 46 |
| 97 | Johann de Vries | Canada | |
| 98 | Yvonne Zarnowski | Canada | |
| 99 | Thanos Zavras | USA | 25 |
| 100 | Maria Teresa Zenzes | Canada | 32 |
| 101 | Vladimir P. Zharov | USA | 40 |
| 102 | Shu-Hong Zhu | USA | 30 |

**Conference Address:**

ISPTID 2003
Department of Oral Biology, Faculty of Dentistry
University of Manitoba, 780 Bannatyne Avenue
Winnipeg, MB, R3E 0W2, CANADA
E-mail: scottda@ms.umanitoba.ca or PTIDSociety@aol.com
Tel: (204) 789-3866 / Fax: (204) 789-3913

**Conference Website:**
www.umanitoba.ca/faculties/dentistry/oral_biology/research/conference

**ISPTID Website:**
www.umanitoba.ca/faculties/dentistry/oral_biology/research/ptid_org

**Tobacco Induced Diseases Website:**
www.umanitoba.ca/faculties/dentistry/oral_biology/research/journal
