# Supplementary material for: Letters of Welcome
Source: Tob Induc Dis. 2003 Dec 15;1(4):229–31. doi: 10.1186/1617-9625-1-4-229 (PMC2672095; doi:10.1186/1617-9625-1-4-229)
Supplement: Additional file 2 [file 1617-9625-1-4-229-S2.pdf]

Conference advertising was generously provided by:

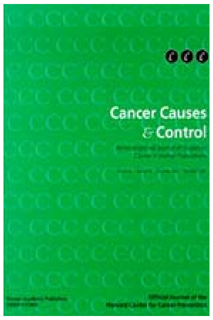

Cancer Causes
and Control

JNCI Journal of the National Cancer Institute

Canadian Journal of Public Health
Revue Canadienne de Santé Publique

Canadian Journal of Physiology
and Pharmacology

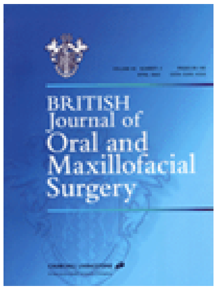

British Journal of Oral
& Maxillofacial Surgery

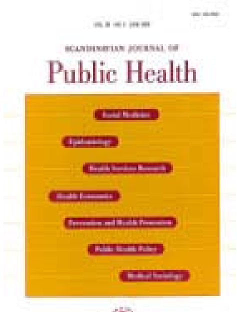

Scandinavian Journal of
Public Health

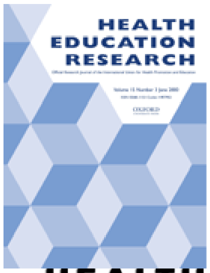

HEALTH
EDUCATION
RESEARCH

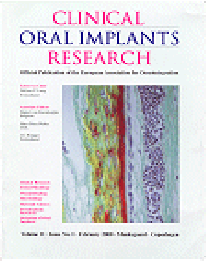

Clinical Oral Implants Research

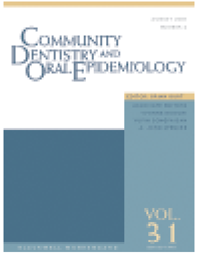

Community Dentistry
& Oral Epidemiology

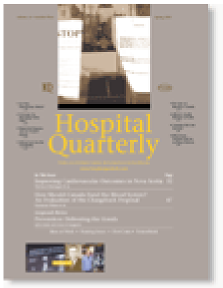

Hospital
Quarterly
